# Effect of morality or capitalist ideology in choosing dentistry as a career in Türkiye: a cross sectional study

**DOI:** 10.1186/s12909-024-05275-8

**Published:** 2024-03-13

**Authors:** Gonca Deste Gökay, Cansu Görürgöz, Ahmet Kılınç

**Affiliations:** 1 Department of Prosthodontics, Faculty of Dentistry, Bursa Uludag University, Bursa, Türkiye; 2 Department of Dentomaxillofacial Radiology, Faculty of Dentistry, Bursa Uludag University, Bursa, Türkiye; 3 Department of Science Education, Faculty of Education, Bursa Uludag University, Bursa, Türkiye

**Keywords:** Career choice, Dental students, Motivation, Altruism

## Abstract

**Background:**

Previous studies have provided important findings on the profession of dentistry and the motivators for choosing dentistry. This study has attempted to contribute to this accumulation by using a sociocultural perspective and interpreting the relationships among motivators from this perspective in a large Turkish sample. The aim of this study was to investigate what motivates dental students to choose dentistry as a career in Türkiye.

**Methods:**

First-year dental students from different dental schools were included in a descriptive cross-sectional study. The questionnaire included sections covering demographics, motivators and career satisfaction. A total of 919 students from 29 different state dental schools and four private dental schools participated. Descriptive statistics such as percentages, means and standard deviations were used to summarize the sociodemographic information and the distribution of the motivators. Factorial analysis was carried out for the structural status of the questionnaire items. The relationships between the motivators were analyzed using Pearson’s correlation.

**Results:**

Among the 919 students, 64.2% were female, and 35.8% were male. Half of them chose dentistry after seeing their higher education institution’s examination results, while the other half had already considered it a career during their preuniversity years. Communicating with people, artistic skills, and helping others were the main motivators for students to choose dentistry as a career in Türkiye, and students’ overall career satisfaction was moderately high. The strongest correlations were between communication with people and benefits (*r* =.74), between communication with people and helping others (*r* =.71), between communication with people and artistic skills (*r* =.66), between artistic skills and benefits (*r* =.69), between artistic skills and helping others (*r* =.65), between dental experience and benefits (*r* =.51), between dental experience and helping others (*r* =.50), and between benefits and helping others (*r* =.71).

**Conclusion:**

Helping others, artistic skills, communicating with people, and benefits were the most important factors motivating dental students to choose a career, and positive relationships were detected between these four factors. This information may help to develop more effective career guidance and mentorship strategies for dental students.

## Background

Morality is based on the distinction between good and bad. Good people desire to develop a good and wealthy civilization by suggesting certain values and beliefs. It determines joint goals for the members of any society, and ‘helping others and protecting them from harm’ is the main driving force underlying these goals. To produce this force, people need the capital from which they can benefit. Money, knowledge and social status/networks are well-known capitals. The main rule within the moral system is to use the capital for one’s own needs first and then share it with others [1].

On the other hand, capitalist ideology is based on a free market and private ownership. Rather than governmental organizations, the private sector decides on economic movements and determines prices according to the interests of customers and competitors. The main idea here is to reach a wealthy society by benefitting from the competitive nature of the market. This system directs people to produce new entrepreneurship opportunities and participate in competitive markets where a range of product alternatives can be produced. Within this economy, people are consumers, and entrepreneurs are trying to understand buying behaviors and to consistently develop products to meet expectations [2].

The production of capital is a difficult task for people because it requires long-term education and nurturing. Throughout the history of human society, people have developed careers in which they not only produce capital but also use it for themselves and others. Within these career systems, people produce, reproduce, transform, and consume capital. For example, as they participate in educational processes, they increase their skills and knowledge. They then convert this knowledge into money and social networks within the same timeframe or in the near future [1]. A career within this economy plays the role of an ecosystem where people can practice their moral values and beliefs. At this point, why one chooses certain careers and how one consumes the capital within the career are crucial moral questions.

It is possible for an individual to desire a particular career for material reasons alone, believing he or she should be able to reproduce the entire capital without sharing those portions that exceed the threshold of need. Conversely, individuals may choose the same career to fulfill altruistic goals as well as material needs. Even if such a simplistic distinction may explain some career choice behaviors, current economic systems and their modification of moral values and beliefs for their own development present a more complex picture [2].

The Career Key is based on John Holland’s theory of career choice. In its simplest form, this theory states that “birds on the same feather were lumped together”. In other words, people like and want to be around people with similar personalities. Career choice means that people choose jobs where they can be around other people like themselves [3].

As a respected and popular profession, dental careers have become increasingly popular with young adults. For the past ten years, dentistry has been listed as one of the best jobs in the U.S. News & World Report’s “Top 100 Jobs” [4]. The U.S. Bureau of Labor Statistics predicts employment growth of 7.6%, equating to 10,400 new dentist jobs through the year 2028 [5]. Although this rate was reported by Marino et al. [6] in 2006 as 65%, 65% of these students did not choose dentistry as their second choice.

Feminization of the dental profession in the last 25 years has been observed in most industrialized countries worldwide. In most European countries and in Türkiye, more than 50% of active dentists are women [7]. Some European countries are unable to reach this rate, but this rate is growing rapidly [8, 9]. At the same time, there is a high proportion of female dentistry students because they believe the profession is more suitable for them.

Previous studies examining the occupational status and educational level of dental students’ parents have shown that most students are from high socioeconomic groups or families with higher education [6, 10, 11]. In one study, most fathers (67.6%) and mothers (51.4%) had an undergraduate or postgraduate education [6]. Regarding ethnicity, Asian fathers are more likely to report an undergraduate or postgraduate education (76.7%) than Australian and international fathers are [6]. According to previous studies, 45% of French students [12] and 60–70% of Australian students [8] lived with their families, so dental students appear to be quite dependent.

Not every high school has an established guidance system that tries to match students’ career choices with their abilities and characteristics. As a result, students may find themselves in a career without knowing why they are being led [13].

The abovementioned knowledge has provided important insights into the profession of dentistry and the motivational factors behind the choice of dentistry. This study attempted to contribute to this accumulation by using a sociocultural perspective and interpreting the relationships between motivators from this perspective.

The purpose of the study was twofold. First, to identify in detail the motivators that influence dental career choice in a large Turkish sample. Second, to identify the nature of the relationships between the motivators. Knowing who and why to choose dentistry as a career can indicate how successful the profession will be in undergraduate education, the quality of service graduates will provide in their professional lives, and the kind of lifelong learning profile they will follow. In other words, it can provide a relatively limited set of information for understanding how a quality dental service is provided. The results of the study may help to develop more effective career guidance and mentoring strategies for dental students.

## Methods

### Study design and population

The study was conducted in 2021 at the Faculty of Dentistry, Bursa Uludağ University, Bursa, Türkiye. The study was approved by the Social Sciences and Humanities Research and Publication Ethics Committee of Bursa Uludağ University, Bursa, Türkiye (reference number: 2020-12-25/2020-10). The empirical research approach involved a quantitative correlational cross-sectional survey design, with data collected through questionnaires and analyzed through descriptive and inferential methods. First-year dental students in Türkiye were included in the study on a voluntary basis. Students who did not want to participate in the study, left the survey questions completely blank and/or left the survey halfway through were excluded.

To determine the survey items, the participants were asked “Why do you want to be a dentist/dental hygienist?” in the Shaikh and Inglehart study [14]. These responses were translated into Turkish by two researchers, and the questions were designed. The suggestions of researchers who are experts in the field of dental education and educational sciences were used in the content validity of the items. The opinions of 10 first-year dental students at Bursa Uludağ University were used for clarity and face validity. A number of items that were difficult to understand were rearranged. A web-based questionnaire was created using Google Forms, and a link to the questionnaire was sent to the dental students.

At the beginning of the survey, participants were told about the study, and their confidentiality was assured. They were informed that we were trying to better understand their motivation for dental education and the factors that influenced their career choices and that their answers would only be used by us. The questionnaire took approximately 15 min to complete. The survey was left accessible online for one month. A total of 919 students from 29 different state dental schools and four private dental schools participated.

### Questionnaire survey

The questionnaire was structured with open-ended and closed-ended questions about who and why they chose dentistry as a professional career. The questions were selected and designed to identify potential motivational factors for choosing dentistry. The final questionnaire consisted of three sections: sociodemographic profile (13 items), motivators for choosing dentistry as a career (55 items), and satisfaction with dentistry as a career (9 items).

Sociodemographic data were collected regarding sex, marital status, parents’ education level, parents’ occupation, selection order for dentistry according to Higher Education Institutions Examination (HEIE) scores, total annual income, and high school graduation. In the motivators section, the students were asked to rate their level of agreement with statements describing various factors of their career choice on a 7-point Likert-type scale (1 = Low effect to 7 = Highly strong effect); this section also included the time spent choosing dentistry as a career option, classified as “after seeing their HEIE results”, “during high school years”, “during primary school years”, “during preschool years” and “other periods”. In the career satisfaction section, a 7-point Likert-type scale (1 = none to 7 = extreme) was used to rate statements about the consistency of the decision to become a dentist.

### Data analysis

All the questionnaires were verified for completeness, and the data were exported from Google spreadsheets. After sorting the data, SPSS version 22 (SPSS, Inc., Chicago, IL/USA) was used for analysis. Descriptive and inferential statistics were calculated.

The skewness and kurtosis scores of the data set were between − 2 and + 2, meaning that the data exhibited a normal distribution [15]. Descriptive statistics were used to summarize the sociodemographic information and the distribution of the motivators. Factorial analysis was carried out for the structural status of the items. The relationships between the motivators were analyzed using Pearson’s correlation.

## Results

### Preliminary analysis

Principal component analysis with varimax rotation was used to determine the factorial structure of the “Motivators for Choosing Dentistry as a Career” (MCDC) and “Dental Career Satisfaction” (DCS) questionnaire items. Due to semantic problems and joint factor loadings, we deleted several items from these questionnaires (3 items in the MCD and 3 in the DCS). Tables 1 and 2 show that this analysis yielded eight factors (1-helping others [Alpha = 0.95]; 2-benefits [Alpha = 0.95]; 3-impact of dental experience [Alpha = 0.90]; 4-influence of others [Alpha = 0.76]; 5-artistic skills [Alpha = 0.92]; 6-better than medicine [Alpha = 0.68]; 7-communication with people [Alpha = 0.92]; 8-HEIE score [Alpha = 0.88]; and one factor (career satisfaction [Alpha = 0.87]) for DCS. The alpha scores for all subquestionnaires ranged from 0.68 to 0.95 for the MCDC, and this value was 0.87 for the DCS.

**Table 1:** Factorial structure and factor loadings (FLs) for the MCDC items

Factors	Item	FL
**Helping others** **α = 0.95**	*Help in diagnosis*	0.78
	*Enable others feel good*	0.78
	*Make oral and teeth health of society better*	0.77
	*Better service to society*	0.77
	*helping others*	0.75
	*Take place in the health/oral health services*	0.74
	*Take place in preventive health care*	0.73
	*Help in healing the inequalities in health services*	0.70
	*Develop facilities of health care*	0.70
	*Create beautiful smiles*	0.69
	*Take place in primary care health service.*	0.68
	*Like medicine*	0.66
	*Wish a career in public service.*	0.65
	*Dentistry health services are insufficient*	0.43
**Benefits** **α = 0.95**	*Good salary*	0.81
	*No financial difficulty*	0.81
	*Respected job*	0.70
	*Adjustable working hours according to personal life*	0.67
	*Regular working hours*	0.67
	*Balance between personal and work life.*	0.64
	*Employment diversity*	0.60
	*Regular job*	0.59
	*Self-employment*	0.56
	*No obligatory service after graduation*	0.56
	*Consistent with personal lifestyle*	0.50
	*Like working environment of dentists*	0.48
**Dental experience** **α = 0.90**	*Impact of my dental treatments*	0.84
	*Impact of successful dentist appointments*	0.80
	*Impact of my dental brace treatment*	0.75
	*Impact of my personal teeth experience*	0.73
	*Impact of my pediatric dentist*	0.67
	*Impact of the dentist that I observed*	0.67
	*Impact of my oral surgeon*	0.61
	*Impact of the dentists that I know*	0.49
	*Impact of the dental research*	0.46
**Influence of others** **α = 0.76**	*Influence of family members*	0.79
	*Influence of friends*	0.77
	*Encouragement of my family members*	0.72
	*Encouragement of my teachers*	0.70
	*Influence of career days*	0.40
**Artistic skills** **α = 0.92**	*Being talented in trim works*	0.79
	*Trust in hand skills*	0.77
	*Wish to become artistic in my career*	0.69
	*Wish to service by an artistic approach*	0.59
**Better than medicine** **α = 0.68**	*Mora advantageous than medicine in pandemic period*	0.66
	*More advantageous than medicine in terms of study duration*	0.66
	*Wish not to have an office job*	0.58
**Communication** **with people** **α = 0.92**	*Being a social person*	0.59
	*Being good in communication with people*	0.56
	*Like communicating with patients*	0.44
**HEIE score** **α = 0.88**	*No option after seeing my HEIE score*	0.92
	*My HEIE score was consistent only with this option*	0.92

**Table 2:** Factorial structure and factor loadings (FLs) for the DCS items

Factor	Item	FL
**Career Satisfaction** **α = 0.87**	*Happy with decision to become dentist*	0.90
	*Suitable job to me*	0.89
	*Career satisfaction*	0.88
	*Desire to change the branch (R)*	0.79
	*Careful thinking about becoming dentist*	0.64
	*Desire to enter HEIE again (R)*	0.61

### Sociodemographic profile of the dental students

In total, 919 students, 590 (64.2%), were female, and 329 (35.8%) were male. In addition, only four (0.4%) students in the sample were married. These results indicate that dental schools are female-dominated learning environments.

A total of 492 (53.5%) students graduated from Anatolian high school, 334 (36.3%) from Science High School, 28 (3%) from schools in other countries, 20 (2.2%) from Open High School, 16 (1.7%) from Basic High School, 13 (1.4%) from Religious High School, 2 (0.2%) from Health Vocational School and 14 (1.5%) from other types of high schools. These results show that the students from the high schools, particularly those offering intensive science and mathematics courses, chose dentistry as a career.

Among the 25 career options available to them according to their HEIE results, 604 (53.6%) students chose dentistry as a career between the 1st and 5th options, 183 (19.9%) between the 6th and 10th options, 84 (9.1%) between the 11th and 15th options, 31 (3.4%) between the 16th and 20th options and seven (0.7%) between the 21st and 25th options. In addition, 441 (47.9%) students chose dentistry as a career after seeing their HEIE results, 403 (43.9%) decided during high school years, 42 (4.6%) decided during primary school years (grades 1 to 8), 4 (0.4%) decided during preschool years, and 29 (3.1%) decided during other periods of their lives (e.g., second year of HEIE preparation). These results show that approximately half of the participants chose dentistry after seeing their HEIE results, while the other half considered it a career during their preuniversity years. In addition, approximately half of the respondents chose dentistry as one of the first five options according to their HEIE scores.

Regarding the educational level of their mothers, 263 (28.6%) had completed primary school, 130 (14.1%) had completed middle school, 249 (27.1%) had completed high school, 251 (27.3%) had completed university, 19 (2.1%) had completed a master’s degree and 7 (0.8%) had completed a doctorate. In terms of occupation, 623 (67.8%) were housewives, 100 (10.9%) worked in the education sector (teachers and academics), 57 (6.2%) worked in the health sector (dentists, doctors, nurses, laboratories, etc.), 43 (4.7%) were laborers, 33 (3.6%) were civil servants, 33 (3.6%) were in commerce and finance, 11 (1.2%) were engineers, 7 (0.8%) were self-employed, 3 (0.3%) were in the security sector and 9 (1%) had a variety of other occupations. These results indicate that mothers have limited education and no or low-paid government jobs.

Regarding the educational level of their fathers, 385 (41.9%) had a university degree, 212 (23.1%) had a high school degree, 136 (14.8%) had a middle school degree, 118 (12.8%) had a primary school degree, 39 (4.2%) had a master’s degree and 29 (3.2%) had a doctorate degree. Regarding occupation, 159 (17.3%) were retired, 134 (14.6%) were workers, 117 (12.7%) were employed in trade and finance, 105 (11.4%) were employed in education, 92 (10%) were civil servants, 78 (8.5%) were self-employed, 60 (6.5%) were in the security sector, 55 (6%) were engineers, 43 (4.7%) were farmers, 40 (4.4%) were in the health sector, and 34 (3.7%) were in a range of other occupations. These results show that fathers had relatively high levels of education and low-paid jobs.

In addition, there were, on average, 4.03 (SD = 1.66, *R* = 0–23) people living in the house. Regarding annual household income, 244 (26.6%) were in the 0–15,000 TL group, 145 (15.8%) were in the 15,001-30000 TL group, 115 (12.5%) were in the 30,001–45,000 TL group, 119 (12.9%) were in the 45,001–60,000 TL group, 89 (9.7%) were in the 60,001–75,000 TL group, 60 (6.5%) were in the 75,001–90,000 TL group, 30 (3.3%) were in the 90,001–105,000 TL group, 55 (6.0%) were in the 105,001–120,000 TL group, and 62 (6.7%) were in the 120,000 + TL group. These results show that they lived in a relatively large family, including at least four other people with low household incomes.

### Comparison of effective motivators

The descriptive results in Table 3 show that benefits (5.07 ± 1.49), communication with people (4.97 ± 1.76), artistic skills (4.74 ± 1.79), and helping others (4.74 ± 1.52) were the main motivators for students to choose dentistry as a career in Türkiye, followed by other motivators such as better than medicine, dental experience, influence of others and the HEIE score. In addition, students’ overall career satisfaction (5.42 ± 1.26) was moderately high.

**Table 3:** Mean (M) and standard deviation (sd) values of MCDC effectiveness and DCS levels

Items	M	sd
*Benefits*	5.07	1.49
*Communication with people*	4.97	1.76
*Artistic skills*	4.74	1.79
*Helping others*	4.74	1.52
*Better than medicine*	3.88	1.78
*Dental experience*	3.14	1.65
*Influence of others*	3.12	1.61
*HEIE score*	2.74	2.08
*Career satisfaction*	5.42	1.26

As shown in Table 4, the Pearson moment correlations between motivators and measures of career satisfaction showed that students who had motivations such as artistic skills, benefits, dental experience, and helping others had higher career satisfaction scores. Similarly, those who considered dentistry to be a career of last resort due to their low HEIE scores were not satisfied with their career choice.

**Table 4:** Correlations between MCDC scores and DCS scores

	Career satisfaction	HEIE score	Communication with people	Better than medicine	Artistic skills	Influence of others	Dental experience	Benefits	Helping others	
Career satisfaction										
HEIE score	**− 0.46**									
Communication with people	**0.28**	0.01								
Better than medicine	**0.13**	**0.09**	**0.42**							
Artistic skills	**0.34**	− 0.04	**0.66**	**0.41**						
Influence of others	**0.13**	**0.11**	**0.39**	**0.37**	**0.35**					
Dental experience	**0.32**	− 0.06	**0.43**	**0.34**	**0.44**	**0.52**				
Benefits	**0.34**	− 0.03	**0.74**	**0.62**	**0.69**	**0.47**	**0.51**			
Helping others	**0.32**	0.06	**0.71**	**0.43**	**0.65**	**0.42**	**0.50**	0.71		

The bold numbers denote *p* <.01

**Fig. 1:**
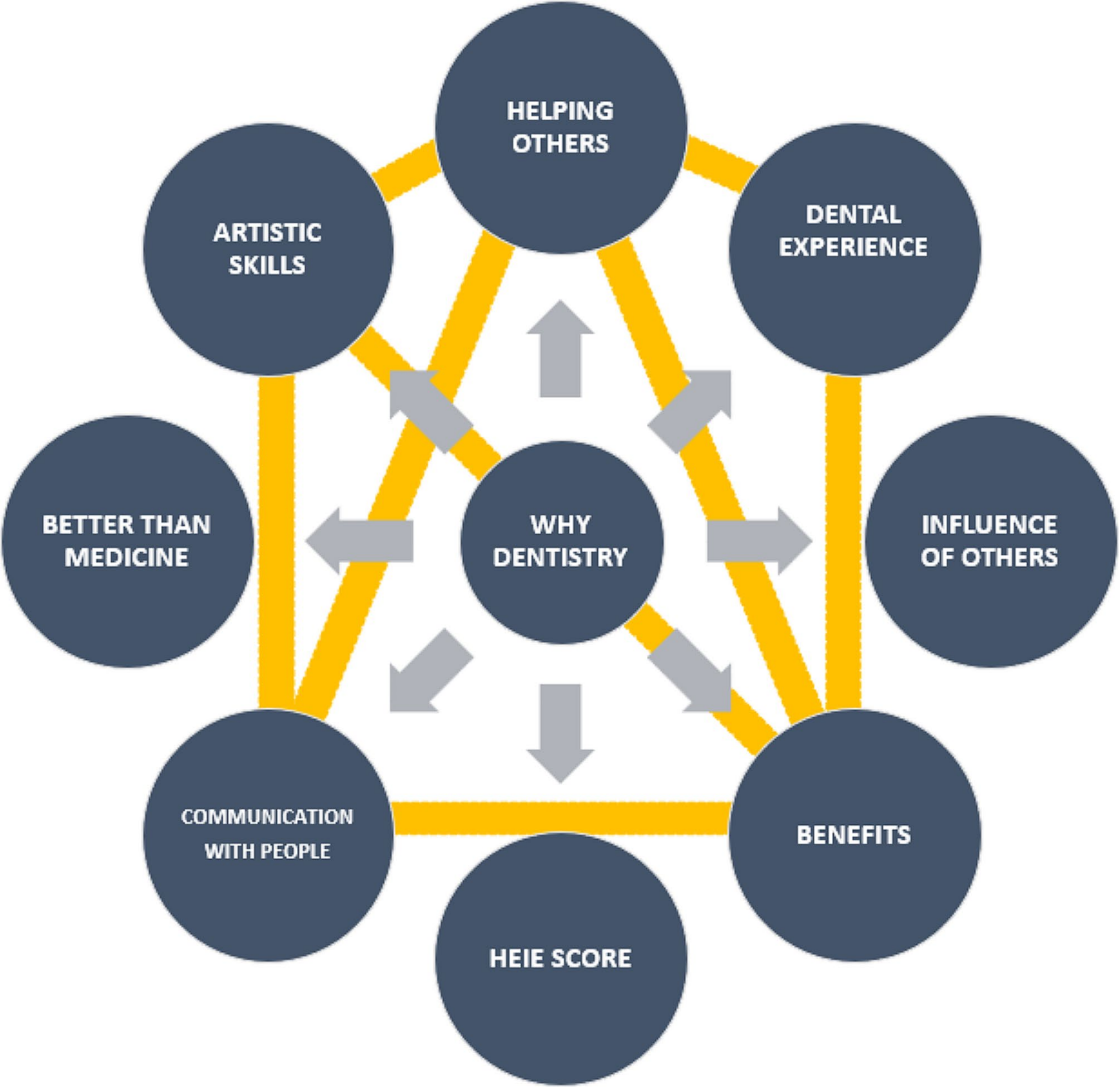
Correlational framework between the motivational factors in dental students

There were also strong positive correlations between most of the motivators (Fig. 1). The strongest correlations were between communication with people and benefits (*r* =.74), between communication with people and helping others (*r* =.71), between communication with people and artistic skills (*r* =.66), between artistic skills and benefits (*r* =.69), between artistic skills and helping others (*r* =.65), between dental experience and benefits (*r* =.51), between dental experience and helping others (*r* =.50), and between benefits and helping others (*r* =.71).

## Discussion

According to the fields of education and training, healthcare programs in Türkiye include dentistry, pharmacy, nursing and midwifery, therapy and rehabilitation, medical diagnostics, and treatment technology and medicine [16]. Respect and prestige are positive features of health professions and have been cited in previous studies as reasons for preferring these professions [17–19].

However, it was also found that dental students’ career choices were motivated by factors other than those of medical students. Among dental students, financial motivations such as high income and financial security were prioritized, and “person-oriented” factors such as “helping people” were less important. Among the motivations of medical students, “financial reward” factors had a lower priority, and “person-oriented” factors were high motivators, with altruism as the primary motivation [20]. It can be predicted that there may be differences in students’ motivation in other health programs. For this reason, the present study focused on the motivators of dental students.

### Sociodemographic profile of the dental students

In the 2020–2021 academic year, 67.4% of the 75,308 students enrolled in health programs were female. Individual factors such as dentistry involving more artistic hand skills, providing more comfortable working conditions, and being one’s own boss can be why female students prefer dentistry. As previous studies on the impact of gender differences on career choice have shown, females were less concerned with the financial component of a career and more concerned with care and human factors [7, 21, 22]. It could be said that females prefer this career because they believe that the nature of dentistry allows them to effectively balance their personal and professional lives [23].

In Türkiye, students must pass a matriculation examination with high marks to be admitted to medical or dental schools. Admission to these schools is based on a centralized examination system conducted annually by the Higher Education Institution, which assesses the student’s knowledge of the basic sciences. There is not yet a national examination, such as one in the United States and Canada, that can be used to assess applicants’ specific skills in attending dental school [22]. As needed by this system, our study showed that students who graduated from high schools offered particularly intensive mathematics and science courses.

Previous studies have shown that most dental students’ parents have a high level of education [6, 10, 11]. Capan et al. [24] reported that 79.1% of fathers and 64.7% of mothers had completed high school or higher. Tanalp et al. [22] reported that 90.4% of fathers and 89.5% of mothers had completed high school or higher. In the present study, the students’ mothers had a low level of education and no or low-paid jobs, while their fathers had a relatively high level of education and low-paid jobs. This difference may be due to social and cultural differences in Türkiye. In the generation of students’ parents who participated in this study, it was common for a man to manage and provide for the family’s needs and financially support the family.

In this study, the majority of dental students lived in large families with limited household income. This finding is similar to that of Marino et al. [6]. In contrast, according to one study, only 24% of French dental students lived with their parents [12]. This may be related to the inability of students to finance accommodations for themselves or their families or to the nature of family relationships in Turkish society. In general, it is traditional for Turkish children to live with their parents until marriage, although there has been a recent trend toward living alone once one has achieved satisfactory economic potential.

There is strong evidence that family income and fathers’ self-employment are important determinants of choosing an occupation with higher labor income risk, such as business, rather than a less risky occupation, such as education or health care, in Türkiye. Poor students may be more likely to avoid risky human capital investments, even if they have high expected personal returns [25]. As a result, students with limited parental income may be more likely to choose careers in education and health. Consistent with these findings, the dental students who participated in the study had a limited income.

### Comparison of effective motivators

It can be argued that the students were aware of crucial humanistic capital, such as knowledge/skills, money/benefits and social relationships/communication with people, given the salient positive correlations between the top four motivators (helping others, artistic skills, communicating with people, and benefits). Students could produce, reproduce, and maintain this capital in this occupation, as in many others. These types of capital are strongly interrelated. For example, skills are essential for earning money and will be useful only in a positive social environment.

Similarly, money requires skills, can be earned through positive relationships, and can only bring happiness by spending with/for others. In regard to helping others, this factor can be assessed in two different dimensions. First, the students seemed to be aware that their future occupation (by nature) was already related to helping people. This situation could be a bonus in addition to increasing capital in their hands. Second, from an altruistic point of view, they may feel that they can use the surplus of their capital to benefit people who need it.

Turkish dental students were influenced mainly by their family/environment [22, 24]. On the other hand, dental experience and the influence of others were not significant motivators for this cohort. Being inspired by a close family member in the same profession may differ from being influenced by friends or teachers. It can be assumed that having close family members in these professions plays an active role in students’ career decisions. This result may be explained by the fact that only 6.2% of the students’ mothers and 3.7% of their fathers work in the health sector. Another explanation may be that students do not pay much attention to their families’ opinions when choosing a career, which may be due to shortcomings in the parent–child relationship or parents’ lack of familiarity with their children’s needs and abilities. These findings are consistent with similar studies in the UK [26] and Iran [27].

Although the sociodemographic profiles of dental students change, the main motivators for choosing a career in dentistry remain similar to those in previous studies [6, 11, 28]. Various motivating factors were determined, including ‘‘helping others’’, ‘‘communication with other people’’, ‘’flexible hours’’, ‘‘financial independence’’, ‘‘financial reward’’, ‘‘prestige’’, ‘‘interest in science’’ and ‘‘parental influence’’ [6, 11, 13, 14, 26–30]. The top motivators in this study were helping others, having artistic skills, communicating with people, and receiving benefits. The results were the same as those of an earlier study that assessed dental students’ career motivations over nine years [14]. Although this study involved first-year students, these top motivators will continue to emerge in the long term, given the research findings above.

The reasons for choosing dentistry as a career differ from country to country. The most common reasons for choosing dentistry were “family expectations” in Japan [29], “income and prestige” in Malaysia [31] and Australia [32], and “helping other people”, which is consistent with the present findings in Sweden [29], Jordan [30], the U.S [14]. and Qatar [33]. A recent study showed that, compared with students with family and environmental factors, most Turkish dental students were self-motivated to choose dentistry as a career [34]. Hatipoğlu revealed that ‘‘income and prestige’’ are the main essential factors in the careers of Turkish dental students [35]. Their career motivations seemed to be related to the socioeconomic aspects of dentistry, in line with the findings of the present study. However, another recent study showed that financial reward was not a motivational factor among dental students [33]. This contradictory result may be because Qatar, where the abovementioned study was conducted, is generally among the 10 richest countries in the world, and the present study included Turkish dental students with low household incomes.

A study of the motivation and confidence of dental students showed that students were disappointed with the program’s content and its integration with the medical curriculum. Many students reported that they had not noticed the breakdown of the didactic program along with the introduction to the dental subjects, that they had changed their minds about their choices and that some were even unhappy with their choices [36]. The element of confidence is essential in career choice and acts as a motivator, enabling students to accept their chosen career with greater satisfaction during difficult times that await them during their academic years. Previous studies have shown that the first choice of dental students is often medicine [18, 37, 38]. This finding was categorized as a healthcare-related factor for positive reasons; however, this difference seemed related to the difficulty of entering medical school in each country. In most countries, admission to medical school is highly competitive, and medicine is usually reserved for high-scoring students; students with lower scores tend to enter dentistry. Tanalp et al. [22] showed that more than half of the students reported dentistry as their first choice in matriculation examinations. Of the ninety-five students whose first choice was not dentistry, fifty-seven (60%) had chosen medicine first, followed by pharmacy and other fields [22]. In a recent study involving 1007 students from Türkiye, 44% of the participants stated that they wanted to study medicine or dentistry, but they chose dentistry because of their HIEI scores. It was determined that the majority of the participants (63.2%) preferred dentistry after medicine or other professions [39]. Similarly, more than half of the students in this study chose dentistry after seeing their HEIE score, and most included it in their top five choices. Dental students’ career satisfaction based on HEIE scores did not meet expectations. Without further evidence, it can be assumed that our dental students did not develop satisfaction with this career because they did not achieve the grades needed to enter medical school.

Dental education is a costly burden for individuals and their communities, especially in countries where education spending is mainly supported by the state, as in Türkiye. Government expenditures per higher education student in Türkiye were $2,961 in 2020 and $3,223 in 2021 [40]. The loss of a dental student after a long and expensive education is a significant loss of resources and a potential lost opportunity for another candidate who may become a more productive member of the dental workforce. The way to prevent these losses is to increase students’ motivation while they are studying dentistry, to have an idea of the extent of their satisfaction, to determine the methods of improvement if the dissatisfaction is based on external factors and to bring the student into the dental workforce.

Identifying the key motivators for why students choose dentistry is important because it may lead to different remedial strategies to support students’ intentions to act and improve educational outcomes. One study has shown that students’ lack of motivation at university has a detrimental effect on their overall mental health and increases their risk of dropping out [41]. A previous study revealed that dental students had more doubts about their choice of specialty during the preclinical years. This doubt decreased as students entered their clinical years [36]. However, a study by Çapan et al. [24] reported that 30.1% of fourth- and fifth-year dental students wanted to change their careers. In a study conducted in three non-European countries, one-third of third-year dental students from Skopje reported that they were not motivated to finish their dental education. It was also found that the greater the number of years of study, the greater the motivation to graduate from school and start working as a dentist [42]. Moderately high levels of career satisfaction were found among our dental students, even though they were in their first year. It can be predicted that the career motivation of our dental students will increase as their clinical practice experience increases and they approach graduation.

The children of the middle class and below in Türkiye wanted to work their way out of the economic maelstrom experienced by their parents, and they wanted to overcome the capital or network problems in their families by turning their knowledge into money. In fact, at least half of the participants stated that they did not think about dentistry until they saw their university exam results. Considering that previous experience and liking for the profession from childhood are very important factors for career satisfaction that persist for many years [43], it is understandable that the participants’ desire to leave the economic vortex by obtaining the highest possible score in the university exam replaced these factors. In the present study, “benefits” motivators were found to be the primary motivators for choosing a career in dentistry, while “goodness” motivators were secondary motivators, which is in line with the results in Türkiye [24, 44] and in different countries worldwide [31, 32]. Although the mechanisms of “benefits” and “goodness” are expected to be in conflict, the fact that these two mechanisms go hand in hand in the minds of dental students shows that the capitalist rush transforms the psychological elements of its own accord.

One limitation of this study was the low response rate of several dental schools, which may have contributed to the nonresponse bias of the dental students. Due to the lack of response, it is likely that students from all the dental schools included in this study were underrepresented. Although this study included data from 29 different dental schools, the results cannot be generalized to dental students across Türkiye. Changing environmental conditions, such as the increasing percentage of dentists working in institutional dentistry, are likely to influence career motivations. Cultural issues are known to vary, and future research could explore cultural influences on career choice in dentistry in different parts of the world. Longitudinal studies are needed to better understand the impact of motivators for choosing dentistry and how students’ motivations change during their education. It may also be worthwhile to compare the motivations of students enrolled in state dental schools with those of students enrolled in private schools.

## Conclusion

In a large sample of first-year dental students in Türkiye, ‘‘interest-based’’ motivators were found to be the primary motivators for choosing a career in dentistry, while “goodness” were secondary motivators. A positive correlation of 0.71 between goodness and interest-based motivators represents seemingly opposite belief systems such as morality and capitalist ideology in dentistry career choice. The well-being motivator ‘‘helping others’’ showed a positive correlation of more than 0.65 with ‘‘communicating with other people’’, ‘‘artistic skills’’ and ‘‘benefits’’. The nature of the job is such that people’s artistic and communication skills and their ability to raise money by helping others are likely to contribute to these findings.

## Data Availability

The datasets used and/or analysed during the current study are available from the corresponding author on reasonable request due to privacy reasons and large data size.

## Abbreviations

HEIE: Higher Education Institutions Examination
MCDC: Motivators for Choosing Dentistry as a Career
DCS: Dental Career Satisfaction
